# Evaluation of EuroSCORE II for elective isolated first time CABG patients

**DOI:** 10.1186/1749-8090-8-S1-P72

**Published:** 2013-09-11

**Authors:** A Khan, V Srivastava, F Mourad, R Richards, A Bose

**Affiliations:** 1 Dept of Cardiothoracic surgery, Blackpool Victoria Hospital, Blackpool, UK; 2 Dept. of Cardiothoracic Surgery, Faculty of Medicine, Ain Shams University, Cairo, Egypt

## Background

EuroSCORE II is proposed to be a better predictor than EuroSCORE I of operative mortality in the current cohort of cardiac surgical patients. The aim of this study was to compare the performance of EuroSCORE II with the original EuroSCORE model for isolated CABG patients.

## Methods

Prospectively collected data from institutional computerised database was interrogated for elective patients undergoing isolated first time CABG between May and Dec. 2013. EuroSCORE II and EuroSCORE I (additive and logistic) were calculated. The performance of each was tested using the Receiving Operating Curve analysis.

## Results

Of a total of 341 patients, 286 (83.9%) were males. The mean age was 65.85 ± 9.9 years. The overall 30 day mortality was 1.47% (5 deaths). The mean EuroSCORE II was 1.3 whereas the mean additive and logistic EuroSCORE I were 3.29 and 3.22 respectively. The area under curve (AUC) for ROC analysis were (in brackets) - for EuroSCORE II (0.68 ± .12), additive EuroSCORE I (0.77 ± 0.08) and logistic EuroSCORE I (0.76 ± 0.09).

## Conclusion

All 3 scores showed moderate predictive ability as demonstrated by the AUC. Mean EuroSCORE II was however closest to the actual observed 30 day mortality.

